# Design of synthetic peptide-based fluorescence probes for turn-on detection of hyaluronan

**DOI:** 10.1007/s44211-023-00491-6

**Published:** 2024-01-12

**Authors:** Xinyu Fan, Yusuke Sato, Yudai Shiraki, Seiichi Nishizawa

**Affiliations:** Department of Chemistry, Graduate School of Science, Tohoku University, Aoba-ku, Sendai, 980-8578 Japan

**Keywords:** Hyaluronan, Peptide probe, Aggregation-induced emission, Detection

## Abstract

Herein, we designed and examined a series of fluorescent peptide-based probes for turn-on detection of hyaluronan (HA), a member of the glycosaminoglycan family. We utilized two kinds of synthetic HA-binding peptides as the binding unit for HA, and each peptide was coupled with three kinds of environment-sensitive fluorophores as the signaling unit. From the examination of the peptides, fluorophores, and the position and number of fluorophore modification, we found that X7 peptide (RYPISRPRKR) labelled with an aggregation-induced emission (AIE) fluorogen, tetraphenylethene (TPE), at the N-terminal (named TPE-X7) did function as a light-up probe for HA. The response of TPE-X7 was highly selective to higher molecular weight HA in comparison with lower ones, having the possible potential for the analysis of HA size. TPE-X7 was also applicable to the quantification of HA in synovial fluids.

**Graphical abstract:**

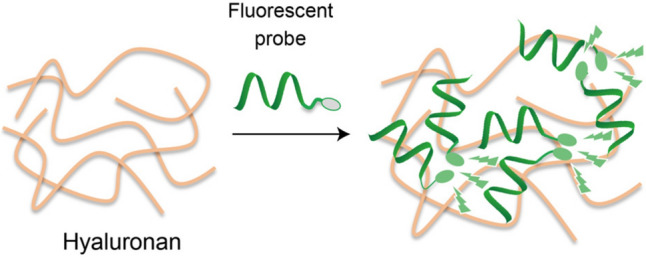

**Supplementary Information:**

The online version contains supplementary material available at 10.1007/s44211-023-00491-6.

## Introduction

**Fig. 1:**
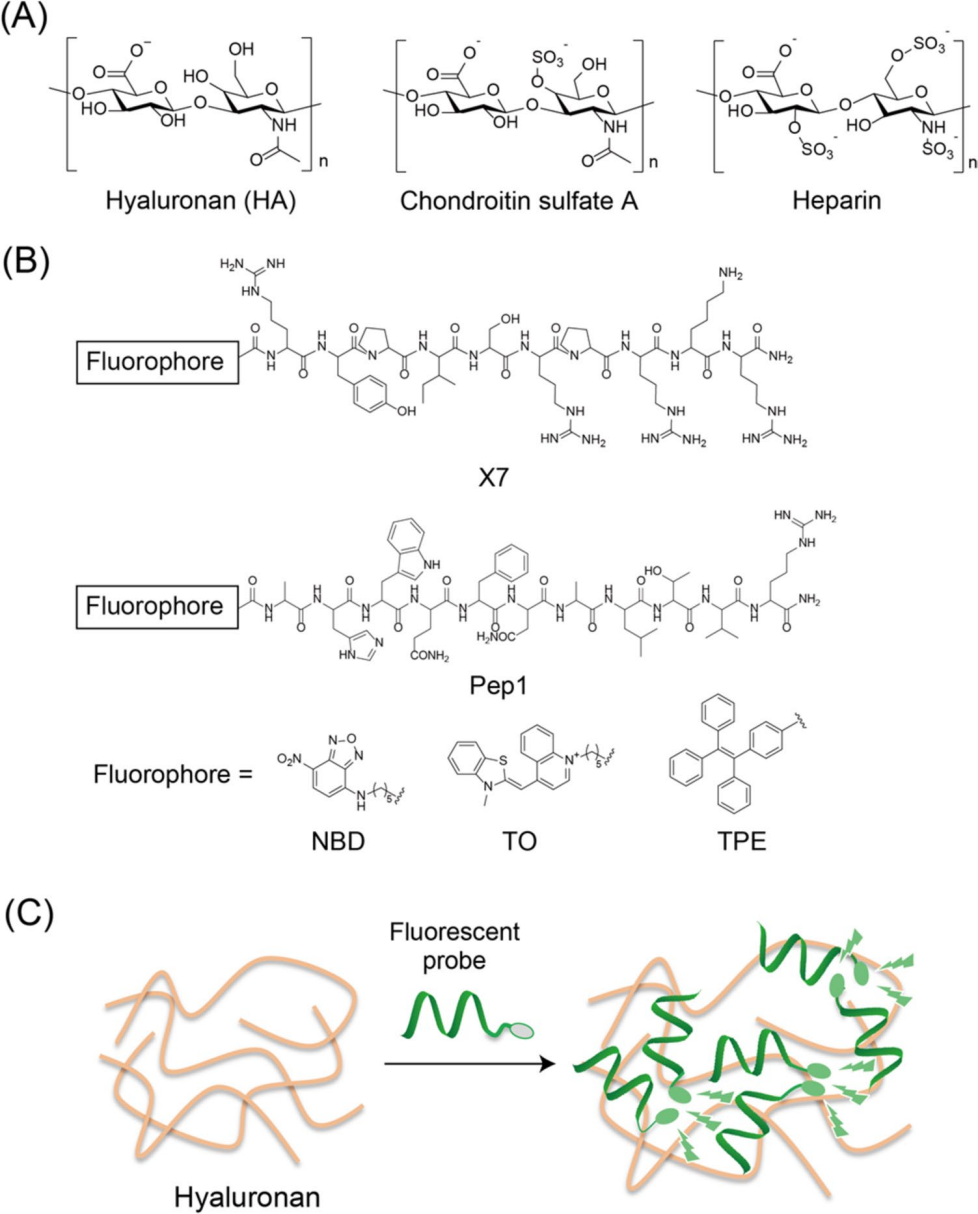
**A** Chemical structures of GAGs. **B** Chemical structures of HA-binding peptide (X7 or Pep1) labelled with fluorophores at N-terminal. **C** Schematic illustration of HA detection based on binding- and aggregation-induced emission of TPE-X7

Hyaluronan (HA), a member of the glycosaminoglycan (GAG) family, is a long unbranched polysaccharide composed of repeating disaccharides of β-D-glucuronic acid and N-acetyl-β-D-glucosamine (Fig. 1A). HA is a major component of the extracellular matrix (ECM) and plays a critical role in biological events, including cell proliferation and differentiation [1, 2]. It is shown that HA is also involved in carcinogenesis, immune cell activation and senescence [3]. Many studies revealed a great potential of HA as a biomarker of pathophysiology and inflammation for diagnostic applications [4]. Currently, the most common method for quantitative detection of HA is enzyme-linked immunosorbent assay (ELISA)-like technique, where HA-binding proteins (HABPs) are utilized as detection agents in competitive and sandwich assays [5, 6]. While HA concentration in the biological fluids can be successfully determined, this method is labor-intensive and time-consuming due to the need of multiple washing steps. Alternatively, much attention has been paid to fluorescent molecular probes capable of binding to GAGs because they enable rapid and simple detection. For example, various kinds of molecular scaffolds including small molecules and peptides have been explored for detection of higher charged GAG, heparin (Fig. 1A), where positively charged aggregation-induced emission (AIE) fluorogens have been utilized for light-up detection [7]. In sharp contrast, the fluorescent probes targeting less charged GAGs such as HA and chondroitin sulfate A (Fig. 1A) have been less developed because of the lack of rational design to achieve selectivity for these GAGs over heparin. Especially, there are very limited reports on the development of fluorescent probes suitable for HA analysis. So far, AIE-based fluorescent probes have been reported for detection of hyaluronidase (not for direct detection of HA), including tetraphenylethene (TPE) derivative (named TPE-4N^+^) [11], pyrene derivative [12], and perylene diimide derivative [13]. Also, an upconversion luminescence nanoprobe has been proposed for sensitive detection of hyaluronidase [14]

In this work, a series of fluorescent peptide-based probes was designed and examined for turn-on detection of HA (Fig. 1B). In contrast to abovementioned AIE-based fluorescent probes for detection of hyaluronidase [11–14], we utilized synthetic HA-binding peptides such as X7 [15, 16] or Pep1 [17] as the probes’ binding units, and then coupled with environment-sensitive fluorophores in order to obtain the fluorescence signals upon binding to HA. We found that X7 peptide labelled with an AIE fluorogen, TPE unit, at the N-terminal (named TPE-X7) served as a useful turn-on probe for HA detection (Fig. 1C). We demonstrated that TPE-X7 was applicable to the quantification of HA in synovial fluids.

## Experimental

### Reagents

Fmoc-protected amino acids with L-configuration except glycine were purchased from Watanabe Chemical Industries (Hiroshima, Japan) or AAPPTec (Louisville, KY, U.S.A.). Hyaluronan from *Streptococcus equi*, chondroitin sulfate A (CS) from bovine tracheae (molecular weight 20–30 kDa), heparin (Hep) from porcine intestinal mucosa (molecular weight 16–17 kDa) were purchased from Sigma-Aldrich (Milwaukee, WI, U.S.A.). Human synovial fluid was purcahsed from BioIVT (Westbury, NY, U.S.A.). The amount of HA in synovial fluid was quantified using a competitive ELISA assay kit (Cosmo Bio Co. Ltd., Tokyo, Japan) accoring to the manufacture’s protocol. HA standard solutions involved in the kit (molecular weight 600–1120 kDa, from rooster comb) were used for construction of the calibration curve. Other reagents were commercially available analytical grade and were used without further purification. All GAGs (glycosaminoglycans) were disooloved in 0.1 M NaCl to obtain 1.0 mg/mL stock solutions. MALDI-TOF-MS spectra were recorded on a Bruker Daltonics autflex Speed-S1 (Bruker, Germany). High-resolution ESI-MS spectra were recorded on a JEOL JMS-T100CS instrument. Water was deionized (≥18.0 MΩ cm specific resistance) by an Elix 5 UV water purification system and a Milli-Q Synthesis A10 system (Millipore Corp., Bedford, MA), followed by filtration through a BioPak filter (Millipore Corp.). All probe stock solutions were prepared using DMSO and were diluted with Milli-Q water for measurements.

Unless otherwise mentioned, all measurements were performed at 25°C in PBS buffer (pH 7.4). Errors are the standard deviations obtained from three independent experiments (*N* = 3).

## UV-visible and fluorescence spectra measurements

Absorption and fluorescence spectra were measured using a JASCO model V-570 UV–vis sectrophotometer and FP-6500 spectrofluorophotometer (Japan Spectroscopic Co. Ltd., Tokyo, Japan), respectively. Both instruments were equipped with thermoelectrically temperature-controlled cell holders. Measurements of absorption and fluorescence spectra were done using a 2 × 10 mm quartz cuvette (optical path length: 10 mm) and a 3 × 3 mm quartz cuvette, respectively. Fluorescence quantum yield (*Φ*) of the probe was determined relative to quinine sulfate (*Φ* = 0.54, in 0.1 M H_2_SO_4_) [18].

## Probe synthesis

All probes were synthesized using Biotage Initiator+ microwave peptide synthesizer (Biotage, Uppsala, Sweden) based on a Fmoc solid phase peptide chemistry on a Rink-Amide-ChemMatrix resin (Biotage). 1-[(1-(cyano-2-ethoxy-2-oxoethylideneamino-oxy)-dimethylamino-morpholino methylene)]methana minium hexafluorophosphate (COMU) / diisopropylethylamine (DIEA) system was employed for the coupling reaction. After completion of the elongation of all amino acid residues, the carboxylate group-carrying fluorophore (4-(1,2,2-triphenylvinyl)benzoic acid, NBD-C4-COOH [19], or TO-C4-COOH [20]) was introduced at N-terminus. The deprotection of the peptide probes and the cleavage from the resin were conducted using trifluoroacetic acid/ m-cresol (95/5). The solution was dropped into cold diethyl ether in order to precipitate the crude peptide probe. The obtained crude product was purified by a reverse-phase HPLC system (pump, PU-2086 Plus ×2; mixer, MX 2080-32; column oven, CO-1565; detector, UV-2070 plus and UV-1570M (Japan Spectroscopic Co. Ltd., Tokyo, Japan)) equipped with a C18 column (Inertsil ODS3 (5.0 μm particle size, 250 × 20 mm column size); GL Sciences Inc., Tokyo, Japan) using a gradient of water/acetonitrile containing 0.2 or 0.1% TFA (Table S1). Absorption at 260 nm, 315 nm, 480 nm, and 510 nm was monitored during HPLC for peptide, tetraphenylethene (TPE) unit, nitrobenzoxadiazole (NBD) unit and thiazole orange (TO) unit, respectively.

## Results and discussion

First, X7 peptide sequence (RYPISRPRKR) [15] was employed for the probe design (Fig. 1B). This peptide contains a well-known HA-binding motif, a B-X_7_-B motif (B: a basic amino acid residue, X: a non-acidic amino acid residue) which is widely found in various HABPs [16]. Indeed, synthetic X7 peptide was shown to bind to HA *in vitro* by resonant mirror biosensor (IAsys) [15]. Here, three kinds of fluorophores, nitrobenzoxadiazole (NBD), thiazole orange (TO) and tetraphenylethene (TPE), were explored as the fluorescent units attached to the N-terminal of X7 due to their potential for turn-on sensing of biomacromolecules by conjugation with peptide scaffolds. It is well established that NBD becomes emissive in the hydrophobic regions [21]. TO has been utilized as it shows off-on response when its torsional motion between the benzothiazole and quinolone rings is restricted in the complex with target molecules [22]. Meanwhile, TPE belongs to the class of aggregation-induced emission (AIE) fluorogens [23]. In contrast to conventional fluorophores with aggregation-caused quenching (ACQ) properties, the emission of TPE is enhanced by the aggregation events. Indeed, TPE-carrying peptide probes were shown to be useful for turn-on detection of heparin [7, 8].

All X7 probes were synthesized by solid phase synthesis based on Fmoc chemistry. The crude product was purified by a reverse-phase HPLC system and the identity was verified by MALDI-TOF-MS (Fig. S1, S2 and Table S1).

**Fig. 2:**
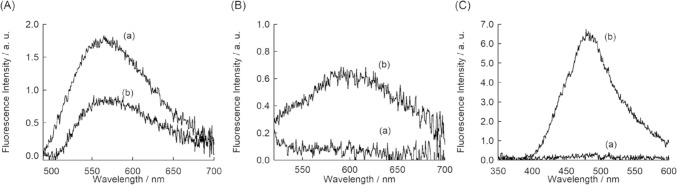
Fluorescence spectra of X7 peptide-based probes (1.0 μM) carrying **A** NBD, **B** TO or **C** TPE in the **a** absence and **b** presence of 0.5 mg/mL HA, measured at 25°C in PBS buffer (pH 7.4). Excitation, **A** 480 nm, **B** 510 nm, and **C** 315 nm

We first measured the fluorescence spectra of three kinds of X7-based probes (1.0 μM) in the absence and presence of 0.5 mg/mL HA from *Streptococcus* *equi* (molecular weight 1500–1800 kDa). As shown in Fig. 2, the fluorescence response for HA significantly depends on the fluorescence signaling unit attached to X7 peptide. NBD-X7 probe exhibited the decrease in the emission for HA, in contrast to our expectation (Fig. 2A). This indicates that NBD unit is not located at the hydrophobic environment for the complexation with HA. On the other hand, TO-X7 and TPE-X7 became emissive in response to HA (Fig. 2B and 2C). Significantly, TPE-X7 showed larger response for HA (29-fold) in comparison with TO-X7 (9.0-fold). As for the TO probe, the observed response (~530 nm) is highly likely due to the restricted rotation of TO unit upon the probe binding to HA [24, 25]. TPE-X7 probe shows the strong emission with maximum at 477 nm for HA, which represents AIE property of the TPE unit in the probe [7, 8]. The TPE units are highly likely to exit in the aggregated states when bound to HA, leading to a turn-on response in the probe for HA (Fig. 1C). This shows the HA acts as a useful aggregation-induced scaffold for TPE-X7 probe, as observed for the TPE derivative (TPE-4N^+^) [11] or a quaternary ammonium-modified TPE derivative [26].

Useful turn-on property of TPE-X7 can be seen from the examination fluorescence quantum yield (*Φ*). While *Φ* value of TPE-X7 was 0.0003 in the buffer, it reaches 0.0052 in the presence of 0.5 mg/mL HA. Importantly, we observed almost no response of a TPE derivative without X7 peptides upon addition of HA under the identical condition (Fig. S3A). This result demonstrates the significant contribution of the X7 peptide to the fluorescence response toward HA. In addition, we compared the fluorescence sensing property of TPE-X7 with that of TPE derivative TPE-4N^+^ (cf. Fig. S3B) that was reported in the literature [11]. This probe was shown to work as the AIE probe for HA upon binding to HA through electrostatic interaction. However, we found TPE-4N^+^ exhibited a minimal response for HA under our experimental condition (Fig. S3B). Compared to such simply modified TPE derivatives, it is likely that TPE-X7 is much more sensitive to HA at least under the present experimental condition.

**Fig. 3:**
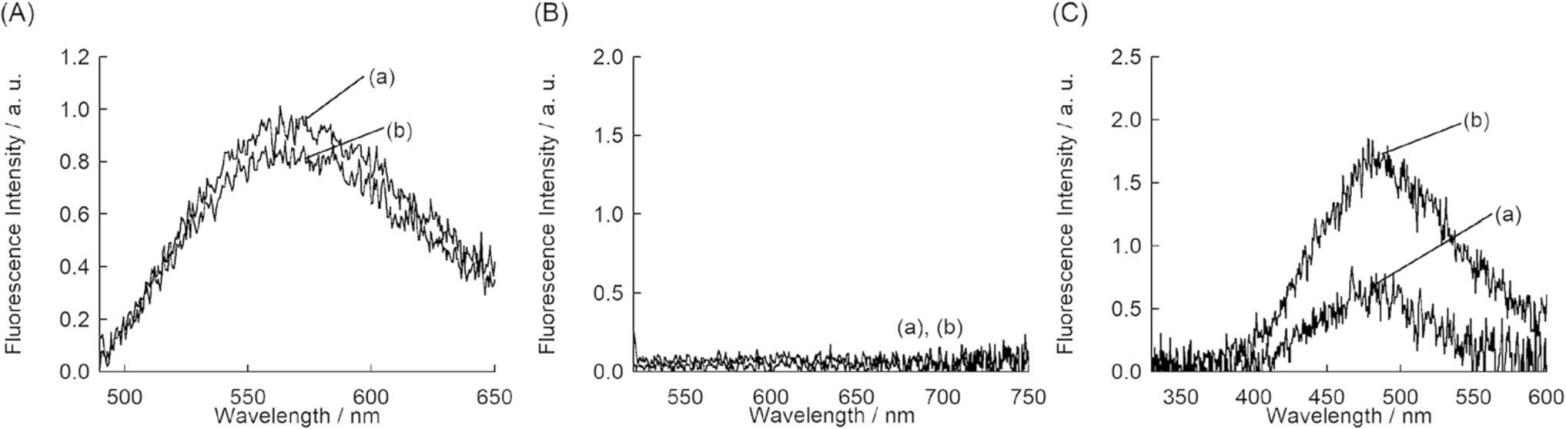
Fluorescence spectra of Pep1 peptide probes (1.0 μM) carrying **A** NBD, **B** TO or **C** TPE at the N-terminal in the **a** absence and **b** presence of 0.5 mg/mL HA. Other conditions were the same as those given in Fig. 2. Excitation, **A** 480 nm, **B** 510 nm, and **C** 315 nm

Such useful response could not be obtained when we explored another HA-binding peptide, Pep1 (12 mer, GAHWQFNALTVR) [17], for the probe design toward HA (Fig. 1B. See also Fig. S1 and Table S1: NBD-Pep1, TO-Pep1 and TPE-Pep1). As observed for X7 probes, TPE-Pep1 exhibited larger increase in the emission compared to NBD- and TO-carrying probes (Fig. 3), but the response of TPE-Pep1 was only moderate compared to that of TPE-X7. Apparently, TPE-X7 does display superior response for HA over TPE-Pep1. These results further support the significant role of the X7 peptide to the fluorescence response toward HA. Also, TPE works well as signaling unit only when it is conjugated with X7 peptide.

Interestingly, the signaling function of X7 probe depends on the position of TPE modification. We prepared X7 probe with TPE unit at the C-terminal (named X7-TPE), for which the additional lysine residue was incorporated at the C-terminal, followed by the coupling of the TPE unit to *ε*-amino group of the lysine residue. Similar to the abovementioned probes with TPE, the fluorescence enhancement based on AIE of TPE unit was observed when the probe binds to HA (Fig. S4A); however, its response (3.6-fold for 0.5 mg/mL HA) was much inferior to TPE-X7 labeled at N-terminal (cf. Fig. 2C). This suggests the efficient aggregation of the TPE unit when it is coupled to N-terminal of X7 peptide compared to C-terminal. In addition, we examined the probe carrying two TPE units at both N- and C-terminals (TPE-X7-TPE). Intriguingly, we observed very moderate response of the probe for HA, where the increase in the emission was less than 1.4-fold (Fig. S4B). This would be due to high background emission of TPE-X7-TPE in the absence of HA, which is attributable to the probe’s aggregation leading to the AIE of the TPE units. From all these results, X7 probe with a single modification of TPE at the N-terminal (TPE-X7) can be quantified as the most promising probe for HA.

**Fig. 4:**
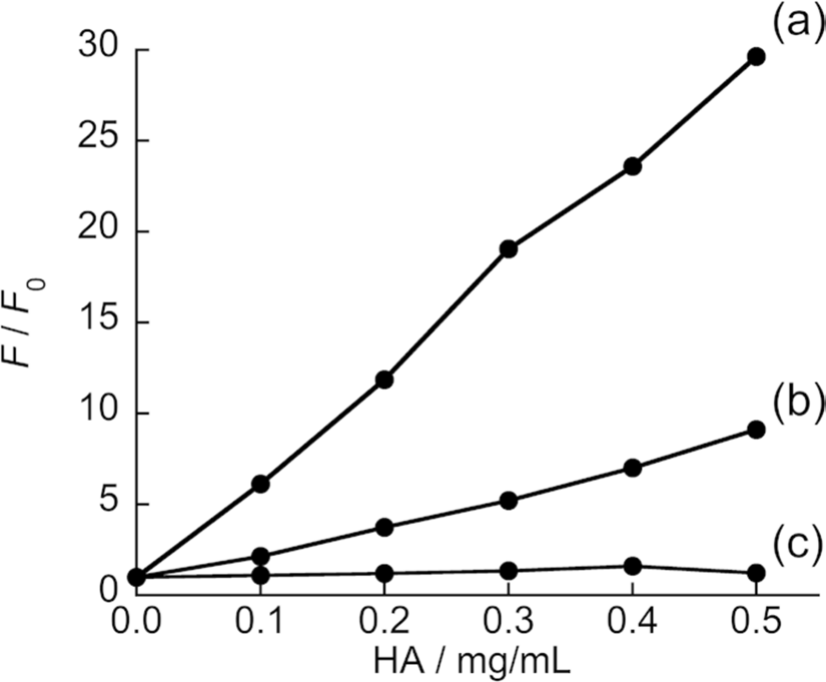
Effect of HA molecular weight (0−0.5 mg/mL; **a** 1500-1800 kDa, **b** 750-1000 kDa, and **c** 150-300 kDa) on the fluorescence response of TPE-X7 (1.0 μM). Other conditions were the same as those given in Fig. 2. Excitation, 315 nm. Analysis, 477 nm

Here, a promising function of TPE-X7 is that its fluorescence response significantly depended on the molecular weight of HA. As shown in Fig. 4, TPE-X7 had negligible emission even in the presence of 150–300 kDa HA. The fluorescence enhancement for 750–1000 kDa HA was observed, but its response was smaller than that for HA with larger MW (1500–1800 kDa). This shows TPE-X7 shows better fluorescence response for higher molecular weight HA in comparison with lower one. This would be due to efficient aggregation of the probe in the complex with HA with longer chain, as can be seen for the heparin-targeting AIE probe [27]. The observed property of TPE-X7 would hold the potential for the analysis of HA size with a view toward the diagnostic applications [3–6]. The limit of detection (LOD) for 1500-1800 kDa HA was determined as 33 μg/mL (*N* = 3) based on 3 times the standard deviation of the blank. The apparent dissociation constant (*K*_d_) for 1500-1800 kDa was estimated as 22 ± 4.9 μM (*N* = 3) by the analysis of the fluorescence titration curve based on a 1:1 binding isotherm [28].

It should be also noted that the fluorescent response of TPE-X7 was significantly reduced to other GAGs with higher charges such as chondroitin sulfate A (charge: -2/disaccharide unit) and heparin (-4/disaccharide unit) (Fig. 1A). The response to HA (charge: -1/disaccharide unit) was indeed comparable to these GAGs (Fig. S5), indicating that the binding of TPE-X7 to GAGs is not governed simply by electrostatic interaction. While further efforts are essential to improve the selectivity toward HA, the signaling function of TPE-X7 for HA is promising when considering that the existing AIE-based probes for GAGs usually show much larger responses to higher charged GAGs [7].

**Fig. 5:**
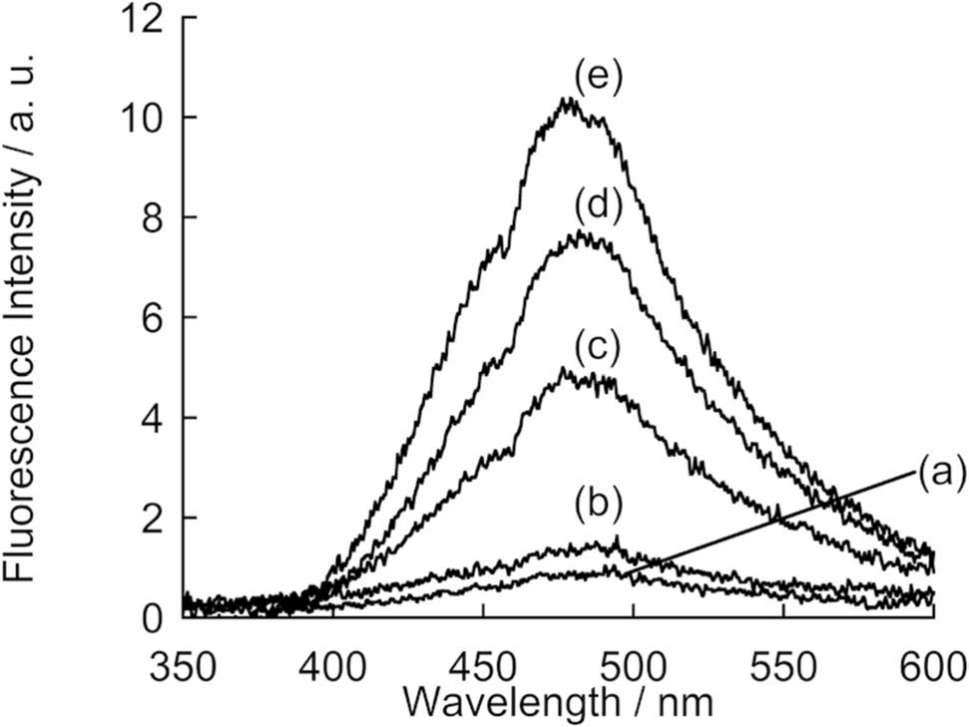
Fluorescence spectra of TPE-X7 (1.0 μM) in the **a** absence and presence of **b** 2000-fold, **c** 200-fold, **d** 20-fold,or **e** 6.7-fold diluted synovial fluid. Other measurement conditions are the same as those given in Fig. 2. Excitation, 315 nm

Actually, TPE-X7 was applicable to the analysis of HA in physiological samples. Here, we focused on synovial fluid, where HA is a major component. Analysis of HA concentration in synovial fluid is of important for diagnostic of osteoarthritis, the most common joint disease [29]. We measured the fluorescence response of TPE-X7 for 2000, 200, 20, 6.7-fold diluted synovial fluid. As shown in Fig. 5, TPE-X7 showed a clear light-up response in the presence of synovial fluid and the response was found to be correlated with the amount of synovial fluid. According to the calibration curve obtained with HA standard solutions, the concentration of HA in the original synovial fluid was determined as 309 ± 50 μg/mL (*N* = 3). This value was in agreement with the results obtained by commercially available ELISA (331 ± 30 μg/mL, *N* = 2). Compared to ELISA that requires a longer assay time (over 3 hours) [30], our assay can be done in a “mix and read” procedure. These results revealed the promising potential of TPE-X7 for the analysis of HA in synovial fluid.

## Conclusions

In summary, we reported on the design of fluorescent peptide probes for turn-on sensing of hyaluronan (HA). X7 peptide (RYPISRPRKR) labelled with tetraphenylethene (TPE) unit at the N-terminal, TPE-X7, showed the light-up response upon binding to HA though aggregation-induced emission (AIE) mechanism. The response of TPE-X7 was highly selective to higher molecular weight HA in comparison with lower ones, and was significantly reduced to other higher charged GAGs such as chondroitin sulfate A and heparin. TPE-X7 would serve as a useful probe for rapid and simple detection of HA, as demonstrated for the quantification of HA in synovial fluids. Meanwhile, further efforts are definitely needed to improve the probe’s functions, including signaling properties, binding affinity as well as binding selectivity toward HA over other GAGs. This would enable the wider applications of the probe for HA analysis in complicated samples containing several kinds of GAGs, such as extracellular matrix. We are now undertaking further studies in these directions.

### Supplementary Information

Below is the link to the electronic supplementary material.

Supplementary file1 (DOCX 2330 KB)

## Data Availability

The datasets generated and/or analyzed during the current study are available from the corresponding authors on reasonable request.
