# Supplementary material for: Design of synthetic peptide-based fluorescence probes for turn-on detection of hyaluronan
Source: Anal Sci. 2024 Jan 12;40(4):609–14. doi: 10.1007/s44211-023-00491-6 (PMC10961276; doi:10.1007/s44211-023-00491-6)
Supplement: Supplementary file 1 — Supplementary file1 (DOCX 2330 KB) [file 44211_2023_491_MOESM1_ESM.docx]

**Supporting Information for**

**Design of synthetic peptide-based fluorescent probes for turn-on sensing of hyaluronan**

Xinyu Fan, Yusuke Sato,* Yudai Shiraki and Seiichi Nishizawa*

*Department of Chemistry, Graduate School of Science, Tohoku University, Aoba-ku, Sendai, 980-8578, Japan.*

Contents:

1. Chemical structures of peptide probes examined in this study (Fig. S1)
2. HPLC profile for the purification of TPE-X7 (Fig. S2)
3. Probe characterization (Table S1)
4. Fluorescence responses of TPE derivatives for HA (Fig. S3)
5. Fluorescence responses of X7 peptide probes carrying a TPE unit at the C-terminal, and two TPE units at both N and C-terminals (Fig. S4)
6. Comparison of fluorescence responses of TPE-X7 for various GAGs (Fig. S5)


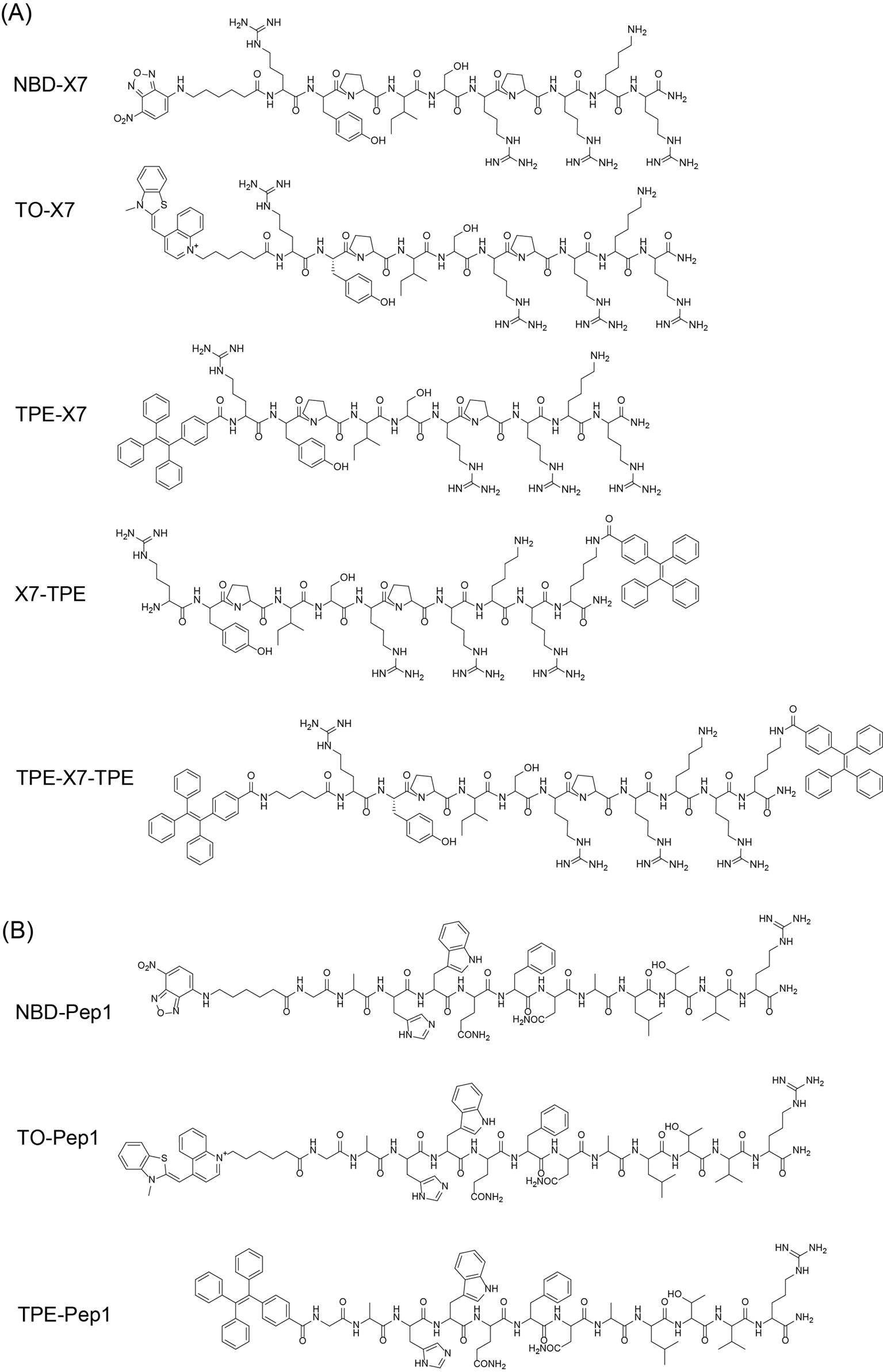


**Fig. S1** Chemical structures of fluorescent probes used in this study.

**
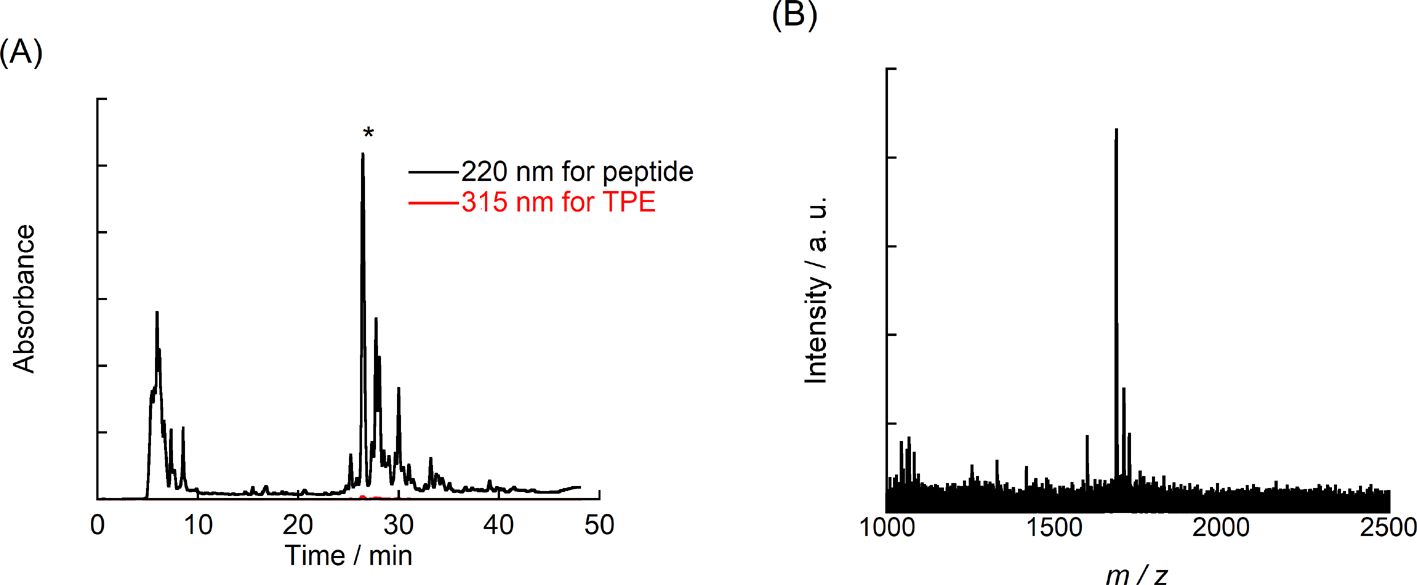
**

**Fig. S2** (A) HPLC profile for the purification of TPE-X7. Gradient condition: 30-60% CH_3_CN (0.2% TFA) in H_2_O (0.2%TFA) during 50min. Absorbance at 220 nm (peptide) and 350 nm (TPE unit) was monitored. The peak (*) was collected and identified as the purified probe. (B) MALDI-TOF-MS spectrum of the purified TPE-X7.

**Table S1** Probe characterization.

|  | Observed mass (m/z) | Calculated mass [M+H]^+^ | Retention time |
| --- | --- | --- | --- |
| NBD-X7 | 1604.27 | 1603.90 | 14 min^(a)^ |
| TO-X7 | 1715.73 | 1714.97 | 7 min^(b)^ |
| TPE-X7 | 1685.67 | 1685.95 | 27 min^(c)^ |
| X7-TPE | 1813.80 | 1814.05 | 5 min^(d)^ |
| TPE-X7-TPE | 2285.27 | 2285.26 | 46 min^(e)^ |
| NBD-Pep1 | 1675.43 | 1674.83 | 5 min^(f)^ |
| TO-Pep1 | 1786.22 | 1785.89 | 12 min^(f)^ |
| TPE-Pep1 | 1757.20 | 1756.87 | 34 min^(g)^ |

Gradient condition: (a) 30-60% CH_3_CN (0.2% TFA) in H_2_O (0.2% TFA) during 32 min, (b) 30-60% CH_3_CN (0.2% TFA) in H_2_O (0.2% TFA) during 40 min, (c) 30-60% CH_3_CN (0.1% TFA) in H_2_O (0.1% TFA) during 40 min, (d) 30-60% CH_3_CN (0.1% TFA) in H_2_O (0.1% TFA) during 50 min, (e) 10-60% CH_3_CN (0.1% TFA) in H_2_O (0.1% TFA) during 60 min, (f) 35-60% CH_3_CN (0.1% TFA) in H_2_O (0.1% TFA) during 40 min and (g) 30-60% CH_3_CN (0.1% TFA) in H_2_O (0.1% TFA) during 40 min.


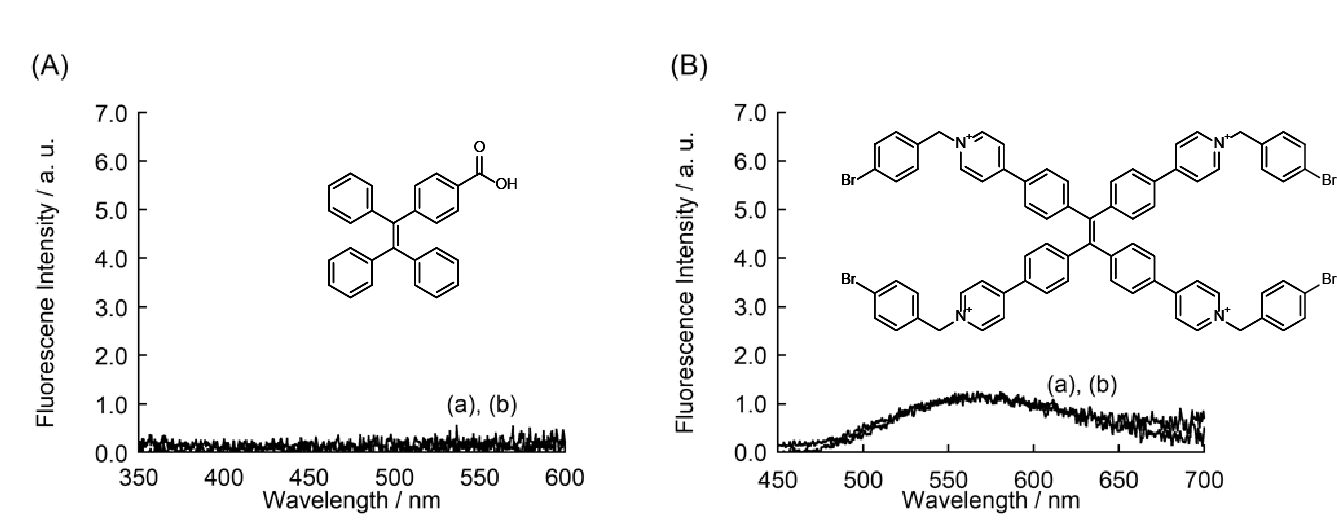


**Fig. S3** Fluorescence spectra of (A) TPE derivative without X7 peptide and (B) cationic TPE derivative [ref. 11 in the main text] in the absence and presence of 0.5 mg/mL HA. Other conditions were the same as those given in Fig. 2 in the main text. Excitation, (A) 315 nm and (B) 348 nm.


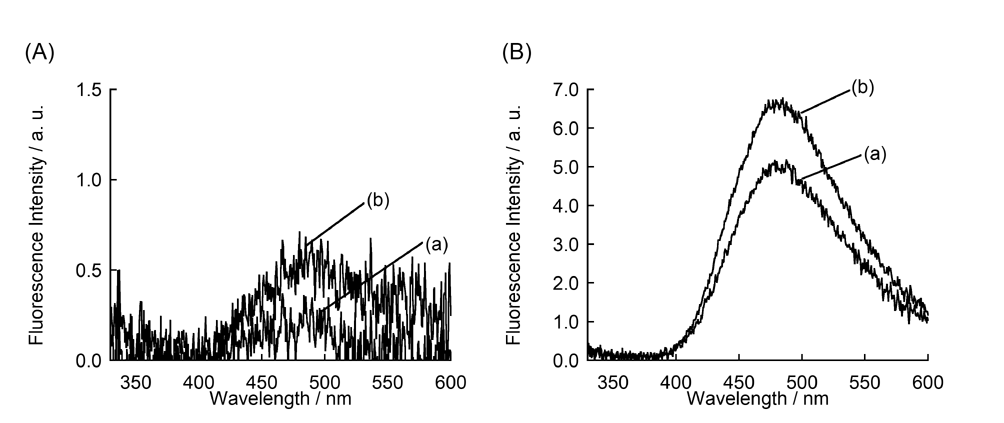


**Fig. S4** Fluorescence spectra of X7 peptide probes carrying (A) a TPE unit at the C-terminal and (B) two TPE units at both N and C-terminals in the (a) absence and (b) presence of 0.5 mg/mL HA. Other conditions were the same as those given in Fig. 2 in the main text. Excitation, 315 nm.


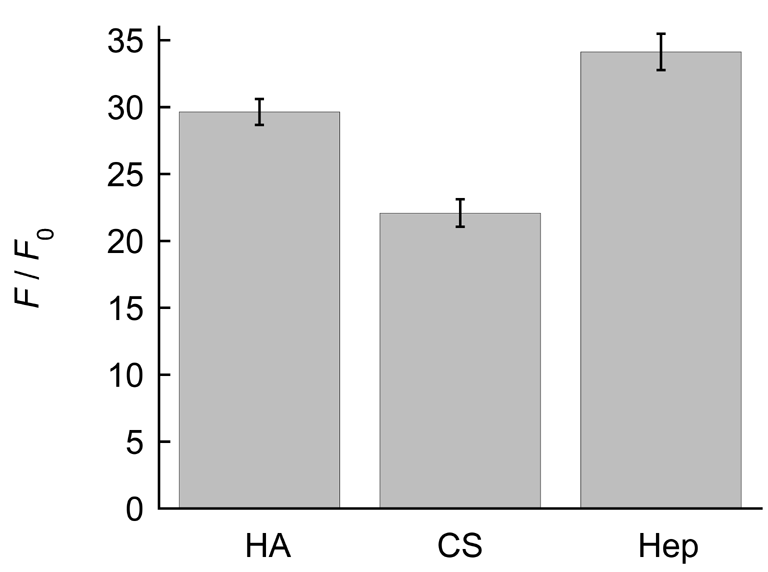


**Fig. S5** Comparison of fluorescence responses of TPE-X7 for various GAGs (0.5 mg/mL: HA, chondroitin sulfate A (CS) and heparin (Hep)). Other conditions were the same as those given in Fig. 2. Excitation, 315 nm. Analysis, 477 nm.
